# Robotic surgery: disruptive innovation or unfulfilled promise? A systematic review and meta-analysis of the first 30 years

**DOI:** 10.1007/s00464-016-4752-x

**Published:** 2016-02-19

**Authors:** Alan Tan, Hutan Ashrafian, Alasdair J. Scott, Sam E. Mason, Leanne Harling, Thanos Athanasiou, Ara Darzi

**Affiliations:** 1 Department of Surgery and Cancer, Imperial College London, 10th Floor QEQM Building, St. Mary’s Hospital, London, W2 1NY UK; 2 Institute of Global Health Innovation, Imperial College London, London, SW7 2NA UK

**Keywords:** Robotic surgery, Conventional surgery, Perioperative outcomes

## Abstract

**Background:**

Robotic surgery has been in existence for 30 years. This study aimed to evaluate the overall perioperative outcomes of robotic surgery compared with open surgery (OS) and conventional minimally invasive surgery (MIS) across various surgical procedures.

**Methods:**

MEDLINE, EMBASE, PsycINFO, and ClinicalTrials.gov were searched from 1990 up to October 2013 with no language restriction. Relevant review articles were hand-searched for remaining studies. Randomised controlled trials (RCTs) and prospective comparative studies (PROs) on perioperative outcomes, regardless of patient age and sex, were included. Primary outcomes were blood loss, blood transfusion rate, operative time, length of hospital stay, and 30-day overall complication rate.

**Results:**

We identified 99 relevant articles (108 studies, 14,448 patients). For robotic versus OS, 50 studies (11 RCTs, 39 PROs) demonstrated reduction in blood loss [ratio of means (RoM) 0.505, 95 % confidence interval (CI) 0.408–0.602], transfusion rate [risk ratio (RR) 0.272, 95 % CI 0.165–0.449], length of hospital stay (RoM 0.695, 0.615–0.774), and 30-day overall complication rate (RR 0.637, 0.483–0.838) in favour of robotic surgery. For robotic versus MIS, 58 studies (21 RCTs, 37 PROs) demonstrated reduced blood loss (RoM 0.853, 0.736–0.969) and transfusion rate (RR 0.621, 0.390–0.988) in favour of robotic surgery but similar length of hospital stay (RoM 0.982, 0.936–1.027) and 30-day overall complication rate (RR 0.988, 0.822–1.188). In both comparisons, robotic surgery prolonged operative time (OS: RoM 1.073, 1.022–1.124; MIS: RoM 1.135, 1.096–1.173). The benefits of robotic surgery lacked robustness on RCT-sensitivity analyses. However, many studies, including the relatively few available RCTs, suffered from high risk of bias and inadequate statistical power.

**Conclusions:**

Our results showed that robotic surgery contributed positively to some perioperative outcomes but longer operative times remained a shortcoming. Better quality evidence is needed to guide surgical decision making regarding the precise clinical targets of this innovation in the next generation of its use.

Robotic surgery represents a fundamental innovation in health care that is designed to enhance the quality of care for patients. Puma 560 was the first surgical robot applied in a clinical setting to obtain neurosurgical biopsies in 1985 [1]. The authors concluded that the robot contributed to improved accuracy. Since then, increasingly advanced surgical robots have been developed to assist in a rapidly expanding range of operative procedures and anatomical targets (Fig. 1). The drivers for continuous innovation stem from the potential to offer greater operative precision that may translate into enhanced clinical outcomes and the accompanying background of corporate revenues within the healthcare technology sector.

**Fig. 1:**
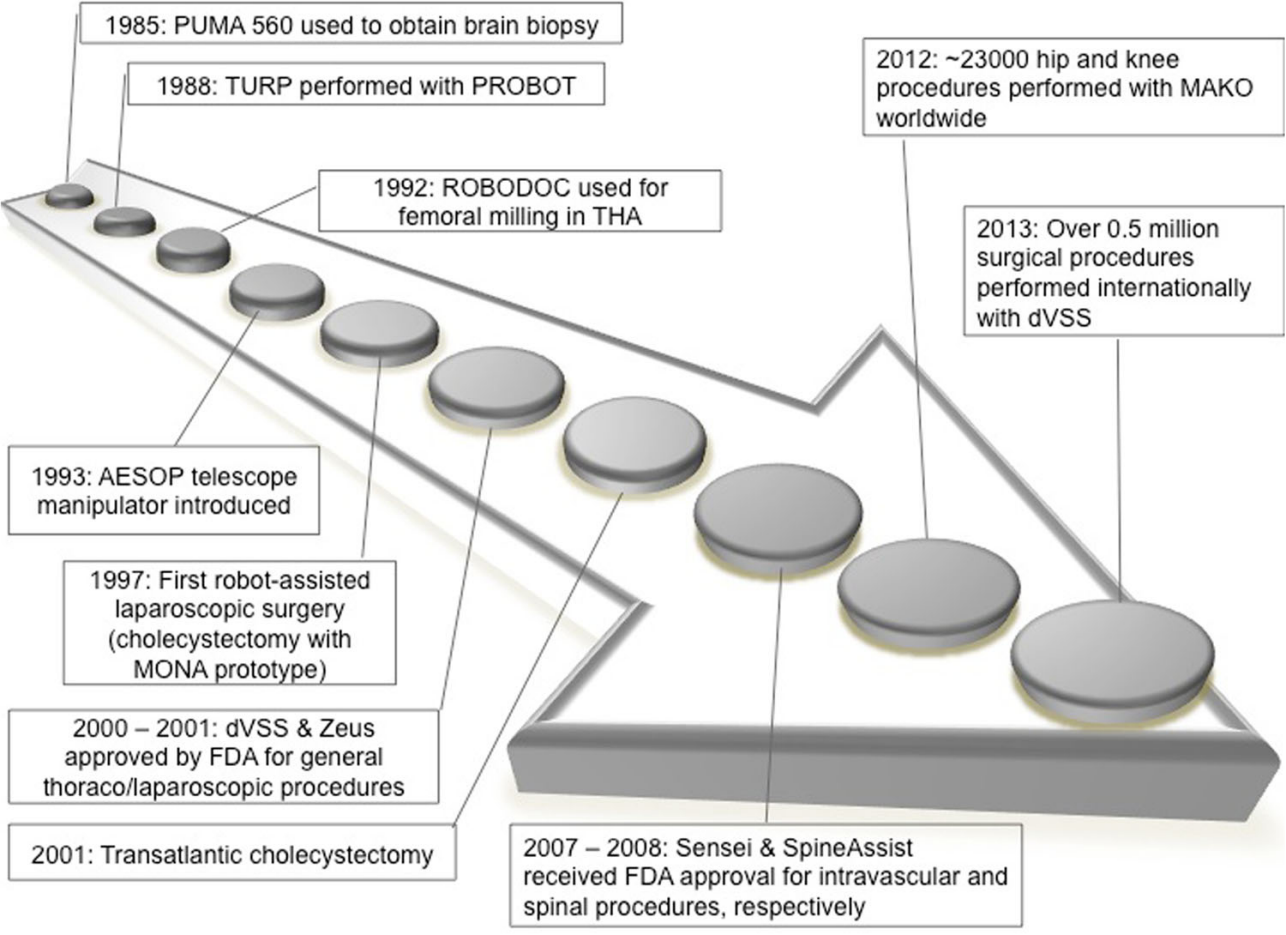
Timeline demonstrating selected events in the history and development of surgical robots

To achieve these goals, current robotic platforms are designed to incorporate advanced features, such as, (i) dexterous capability with accompanying instrumentation, (ii) augmented visualisation, (iii) improved stability, (iv) natural coordination, (v) accurate cutting capacity, (vi) reliable execution, and (vii) enhanced surgeon ergonomics. These features can theoretically increase surgical precision by rendering difficult operative tasks easier to perform safely. Moreover, surgical robots have retained the capacity to enable surgery through smaller incisions. Collectively, these characteristics aim to enhance outcomes beyond that achievable through conventional operative methods.

The adoption and diffusion of robotic surgery demonstrate a positive trend in some geographical areas, particularly for advanced economies. This can be illustrated by the prominent application of the da Vinci^®^ Surgical System (dVSS; Intuitive Surgical Inc., Mountain View, Sunnyvale, California, USA), which has US Food and Drug Administration (FDA) clearance across a multitude of specialties [2], demonstrating its greatest exposure for urological and gynaecological procedures [3]. For example, more than half of radical prostatectomies and about a third of benign hysterectomies are already performed robotically in the USA [3, 4].

Despite offering some elements of innovative technology, the necessary evidence to justify the expanding investment in robotic surgery remains ambiguous. Whilst the concept of robotic surgery is almost universally favoured, its widespread promotion across all healthcare sectors requires robust justification, not least because it can be very costly [5]. Studies comparing outcomes of robotic surgery with conventional approaches for specific robots and procedures are certainly not scarce. However, the systematic assessment of robotic surgery collectively as a single entity has not been performed. As we approach the end of the third decade following the pioneering use of the first surgical robot, an overview of this innovation may be useful for understanding the adoption of innovations in health care.

The aim of this comprehensive systematic review and meta-analysis was to draw evidence from comparative studies in robotic surgery, regardless of specialty and procedure type, and irrespective of patient age and sex. We avoided the biases of retrospective studies that dominate the literature by focussing only on randomised controlled trials (RCTs) and non-randomised prospective studies. In comparing potentially very heterogeneous studies, we emphasised a methodology that identified the proportional benefit of robotic surgical outcomes compared with controls in each study. This offered internal consistency from each study. We were then able to calculate a pooled proportional benefit for specific robotic surgical outcomes for all studies.

In this review, we evaluated core perioperative variables as our primary outcomes. These were (i) blood loss, (ii) blood transfusion rate, (iii) operative time, (iv) length of hospital stay, and (v) 30-day overall complication rate. In robotic surgical studies, these perioperative variables were most commonly addressed. Analyses were performed separately for robotic versus open surgery (OS) and robotic versus minimally invasive surgery (MIS). As a secondary outcome, we calculated the proportion of studies that demonstrated adequate statistical power for the evaluation of these clinical outcomes.

## Materials and methods

This review was performed according to the Preferred Reporting Items for Systematic Reviews and Meta-analyses (PRISMA) statement [6].

### Inclusion and exclusion criteria

We defined surgery as any interventional procedure involving alteration in anatomy and that either requires a skin (or mucosal) incision or puncture. Patients requiring surgery for which a robotic approach was a feasible alternative approach to OS or MIS were included. There was no age or sex restriction. Controls were eligible only if patients underwent surgery and no robotic assistance was provided. RCTs and prospective studies that addressed one or more core perioperative surgical outcomes (blood loss, blood transfusion rate, operative time, length of hospital stay, and 30-day overall complication rate) were included. For operative time, we included studies that explicitly defined it as starting from skin incision to skin closure (for intravascular procedures, we used procedure time, which was generally defined as time from first venous puncture to sheath withdrawal at the end of the procedure). Whilst this measure does not represent the total theatre occupation time, it was selected to improve comparability because operative time was variedly defined in the literature.

We excluded studies where surgical robots were used for stereotactic, endoscopic, or single-incision laparoscopic surgery. Robotic instrument positioners without concurrent use of other robotic instrumentation tools were also excluded, as were innovations that are generally not considered robotic technology, such as remote magnetic catheter navigation and pure computer navigation systems. We also discounted studies with historical controls that preceded the robotic arm considerably (that is, greater than a year) as well as those that retrospectively reviewed and analysed prospective databases. Laboratory studies involving synthetic models, animals, or cadavers were not considered.

### Search methodology

Using the OvidSP search engine, the MEDLINE, EMBASE, and PsycINFO databases were searched on 2 September 2013 with the terms: robot* (tw) AND [intervention* (tw) OR surg* (tw)]. The same search terms were used to search the ClinicalTrials.gov registry to identify potentially relevant trials. On 26 May 2014, these trials were reviewed to identify any relevant published data. To avoid losing generally older papers which had used the term computer-assisted instead of robot, we also performed a search on 7 October 2013 with the terms: [surgery, computer-assisted (MESH, exp) OR computer-assisted surg* (tw) OR computer-aided surg* (tw)] AND [intervention* (tw) OR surg* (tw)]. Studies from 1990 to the search dates were included. There was no language restriction. Relevant review articles, including health technology assessments, found through our search strategy were also hand-searched to identify any remaining studies.

### Data collection and analysis

#### Study selection

Articles were screened from titles and abstracts by three authors independently (AT, SM, and AS). Potentially relevant articles that appear to fit the inclusion and exclusion criteria were obtained in full text. These were independently assessed for eligibility by the same authors. Articles were excluded if they had duplicate or incomplete data, or if they were only available in abstract form. Any disagreement was resolved through discussion with a senior author (HA).

#### Dealing with duplicate publications

If several articles reported outcomes from a single study, the article with the most comprehensive results (most number of patients and/or most recent publication) was included. If this article failed to report outcomes that were otherwise available in the duplicate article, then the additional data from the duplicate article were included.

#### Data extraction

One author (AT) extracted data into an Excel 2011 database (Microsoft Corp., Redmond, Washington, USA), which were then reviewed independently by three authors (SM, AS, and HA). For each article, the year of publication, study design, total number of patients, number of patients in each arm, robot and control type, baseline characteristics, and results of outcome measures of interest were extracted. For continuous outcomes, we extracted the mean and standard deviation (or if unavailable, the median and standard error, range, or interquartile range). For categorical outcomes, we recorded the number of events.

#### Risk of bias assessment

Three authors (AT, SM, and AS) independently assessed the risk of bias of eligible articles. Quality of articles with more than one study was assessed on their overall methodology. The Cochrane risk of bias tool [7] was applied to RCTs. Seven key domains were assessed: method of random sequence generation, allocation concealment, blinding of participants and personnel, blinding of outcome assessors, completeness of outcome data, selective reporting, and other potential sources of bias. Based on a set of listed criteria, each domain was judged to have either a low, high, or unclear risk of bias. If a study had unclear or high risk of bias for one or more key domains, then it was classified as having, respectively, an unclear or high risk of bias overall. If instead all the key domains had low bias risk, then the study was judged to have a low risk of bias overall [7].

For prospective studies, the Newcastle–Ottawa scale (NOS) [8] was used for quality scoring. The NOS judges studies on three categories: the selection of the study groups (comprising four numbered items: representativeness of exposed cohort, selection of non-exposed cohort, ascertainment of exposure, demonstration that outcomes were not present at start of study), the comparability of the groups (comprising one numbered item: comparability of cohorts on basis of study design or analysis), and outcomes (comprising three numbered items: assessment of outcome, appropriateness of length of follow-up, adequacy of follow-up of cohorts). From a set of listed criteria, a maximum of one star can be awarded for each numbered item, except for comparability where a maximum of two stars can be awarded. The possible NOS score ranges from 0 to 9 stars. We classified studies with ≥7 stars as “higher” quality and <7 stars as “lower” quality.

Risk of bias assessment was made at the level of outcomes. We assessed perioperative outcomes together as a class [7, 9]. If a study addressed several perioperative outcomes, the risk of bias for a particular domain was judged based on the outcome that was most affected by the study methodology. Any disagreement with risk of bias assessment was resolved through discussion with a senior author (HA).

#### Data synthesis and statistical methods

Meta-analysis was based on control type, that is, either robotic versus OS or robotic versus MIS. Wherever possible, we used results from intention-to-treat analyses. Continuous outcomes were analysed by calculating the ratio of means (RoM) for each study, with expression of uncertainty of each result represented by the 95 % confidence intervals (CI) [10]. We substituted median for mean in studies where only the median was reported. When the calculated RoM was 1, computation was not possible. Consequently, these results were excluded. Categorical outcomes were analysed using risk ratio (RR) with 95 % CI [7]. Studies reporting categorical outcomes with no events in both the robotic and control groups were excluded, as their effect sizes were not computable. We performed meta-analysis if two or more separate studies were available. The inverse-variance, random-effects model of DerSimonian and Laird [11] was used for both continuous and categorical outcomes. This was accomplished using Stata 13 (StataCorp., College Station, Texas, USA). Sensitivity analysis on RCTs was also performed. The *I*^2^ statistic was used to estimate the degree of heterogeneity between studies, where larger values indicate increasing heterogeneity [12].

*Post hoc* power analysis (significant at the 5 % level, two-tailed t test) was conducted for all eligible studies using the G*Power 3.1 programme [13]. Power was calculated for large (*d* = 0.8), medium (*d* = 0.5), and small (*d* = 0.2) effect sizes. We defined adequate statistical power as >80 %. We also identified studies with clearly specified primary outcomes and where power analysis was performed to determine the required sample size for adequate assessment of these outcomes.

## Results

### Search results

**Fig. 2:**
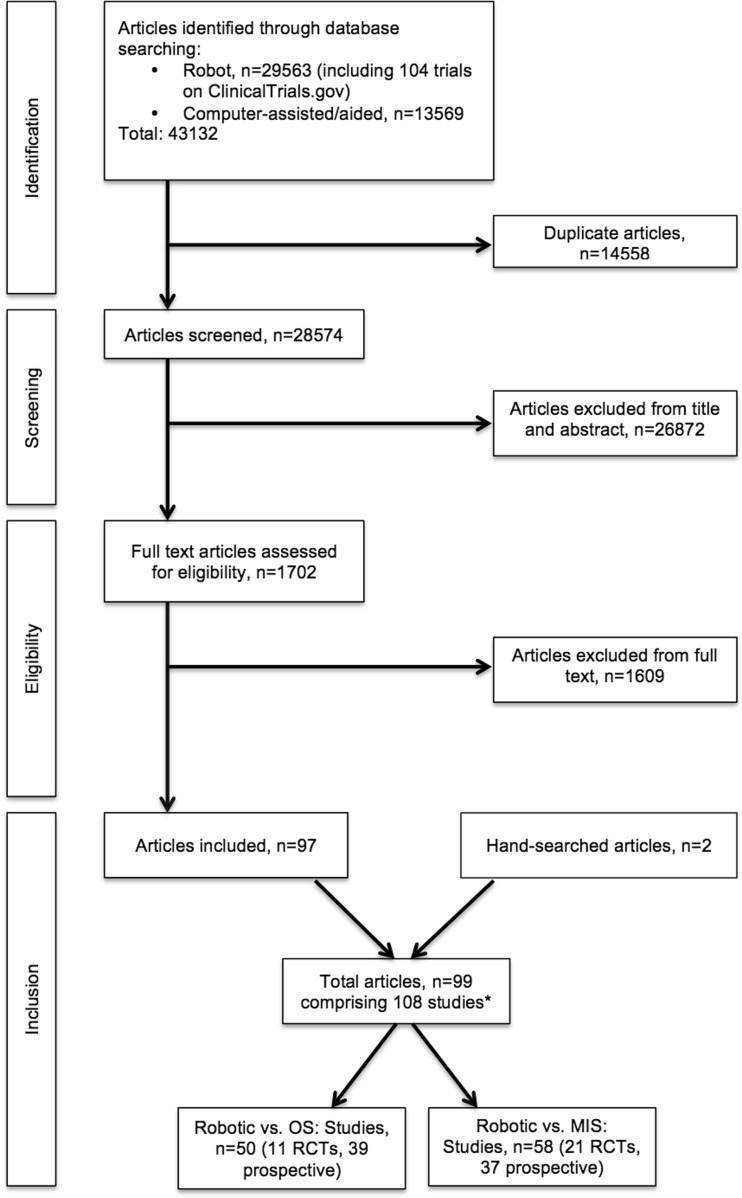
Flow chart of included studies. *Some articles contained more than one comparison or study (see text). *OS* open surgery, *MIS* minimally invasive surgery, *RCT* randomised controlled trial

A total of 43,132 articles were identified from the databases. This included 104 trials from the ClinicalTrials.gov registry, of which one [14] was subsequently found to contain relevant published data. After removing duplicates, 28,574 articles were screened based on their titles and abstracts. Of these, 1702 potentially relevant full-text articles were retrieved for further evaluation. We found 97 articles that met the inclusion criteria. Two additional articles were identified through hand-searching. In total, 99 articles, involving 14,448 patients overall, were included in this review (Fig. 2).

### Description of included studies

Of the included articles, all but one [15] investigated outcomes in adult patients. Overall, there were 31 and 68 articles, respectively, that were based on RCT and non-randomised prospective comparative designs. They encompassed a wide range of specialties and procedures (Tables 1, 2). Some articles comprised more than one comparison or study [16–23].

**Table 1:** Studies comparing robotic versus open surgery

References	Procedure	Design	No. of patients, *n*			Robot	Perioperative outcomes addressed^a^	Power		
			Total	R	C			Effect size		
								Large	Medium	Small
Bertani et al. [16]^b^	Rectal resection	PRO	86	52	34	dVSS	BL, LOS, C	0.948	0.611	0.146
Kim et al. [68]	Rectal resection	PRO	200	100	100	dVSS	BT, LOS, C	1.000	0.940	0.291
Bertani et al. [16]^b^	Colectomy	PRO	79	34	45	dVSS	BL, LOS, C	0.935	0.584	0.140
Lee et al. [47]	Thyroidectomy	PRO	84	41	43	dVSS	BL, OT, LOS, C	0.952	0.619	0.148
Kim et al. [48]	Thyroidectomy	PRO	37	19	18	dVSS	BL	0.657	0.315	0.091
Ryu et al. [96]	Thyroidectomy	PRO	90	45	45	dVSS	LOS, C	0.964	0.738	0.180
Menon et al. [34]	Prostatectomy	PRO	60	30	30	dVSS	BL, BT, OT, LOS, C	0.861	0.478	0.119
Tewari et al. [36]	Prostatectomy	PRO	300	200	100	dVSS	BL, BT, OT, LOS, C	1.000	0.983	0.370
Farnham et al. [31]	Prostatectomy	PRO	279	176	103	dVSS	BL, BT	1.000	0.980	0.362
Wood et al. [35]	Prostatectomy	PRO	206	117	89	dVSS	BL, BT, LOS, C	1.000	0.943	0.293
Nelson et al. [95]	Prostatectomy	PRO	1003	629	374	dVSS	LOS, C	1.000	1.000	0.864
Ham et al. [37]	Prostatectomy	PRO	298	188	110	dVSS	BL, LOS, C	1.000	0.986	0.383
Ficarra et al. [32]	Prostatectomy	PRO	208	103	105	dVSS	BL, BT, LOS, C	1.000	0.948	0.300
Carlsson et al. [111]	Prostatectomy	PRO	1738	1253	485	dVSS	C	1.000	1.000	0.962
Hong et al. [38]	Prostatectomy	PRO	51	26	25	dVSS	BL, BT	0.799	0.417	0.108
Doumerc et al. [69]	Prostatectomy	PRO	714	212	502	dVSS	BT, LOS, C	1.000	1.000	0.684
Kordan et al. [33]	Prostatectomy	PRO	1244	830	414	dVSS	BL, BT	1.000	1.000	0.913
Di Pierro et al. [70]	Prostatectomy	PRO	150	75	75	dVSS	BT, C	0.998	0.860	0.229
Kim et al. [112]	Prostatectomy	PRO	763	528	235	dVSS	C	1.000	1.000	0.721
Ludovico et al. [39]	Prostatectomy	PRO	130	82	48	dVSS	BL, LOS, C	0.992	0.780	0.194
Rhee et al. [42]	Cystectomy	PRO	30	7	23	dVSS	BL, BT, OT, LOS	0.432	0.201	0.073
Nix et al. [25]	Cystectomy	RCT	41	21	20	dVSS	BL, LOS, C	0.704	0.345	0.096
Ng et al. [41]	Cystectomy	PRO	187	83	104	dVSS	BL, OT, LOS, C^c^	1.000	0.922	0.272
Martin et al. [40]	Cystectomy	PRO	33	19	14	dVSS	BL, LOS	0.595	0.280	0.085
Khan et al. [17]^b^	Cystectomy	PRO	100	48	52	dVSS	BL, BT, OT, LOS, C	0.977	0.696	0.167
Parekh et al. [26]	Cystectomy	RCT	40	20	20	dVSS	BL, BT, OT, LOS, C	0.693	0.338	0.095
Masson-Lecomte et al. [49]	Nephrectomy	PRO	100	42	58	dVSS	BL, BT, OT, LOS, C	0.974	0.686	0.165
Parekattil et al. [23]^b^	Vasovasostomy	PRO	94	66	28	dVSS	OT	0.939	0.592	0.142
Parekattil et al. [23]^b^	Vasoepididymostomy	PRO	61	44	17	dVSS	OT	0.787	0.406	0.106
Bucerius et al. [18]^b^	CABG	PRO	117	24	93	dVSS	LOS	0.934	0.581	0.139
Kiaii et al. [94]	CABG	PRO	100	50	50	ZRSS	LOS, C	0.977	0.697	0.168
Poston et al. [43]	CABG	PRO	200	100	100	dVSS	BL, LOS, C	1.000	0.940	0.291
Bachinsky et al. [71]	CABG	PRO	52	25	27	dVSS	BT, LOS	0.807	0.424	0.109
Balduyck et al. [110]	Anterior mediastinal mass resection	PRO	36	14	22	dVSS	C	0.623	0.295	0.088
Hoekstra et al. [20]^b^	Endometrial cancer staging	PRO	58	32	26	dVSS	BL, OT, LOS, C	0.846	0.461	0.116
Göçmen et al. [45]	Endometrial cancer staging	PRO	22	10	12	dVSS	BL, BT, OT, LOS, C	0.428	0.199	0.073
Jung et al. [19]^b^	Endometrial cancer staging	PRO	84	28	56	dVSS	BT, OT, LOS, C	0.927	0.569	0.137
Lowe et al. [46]	Hysterectomy—cervical cancer	PRO	14	7	7	dVSS	BL, OT, LOS, C	0.281	0.139	0.064
Collins et al. [44]	Sacrocolpopexy	PRO	48	30	18	dVSS	BL	0.748	0.375	0.101
Bargar et al. [28]	Total hip arthroplasty	RCT	136	70	66	ROBODOC	BL, LOS, C	0.996	0.825	0.212
Bach et al. [109]	Total hip arthroplasty	PRO	50	25	25	ROBODOC	C	0.791	0.410	0.107
Honl et al. [76]	Total hip arthroplasty	RCT	141	61	80	ROBODOC	OT, C	0.997	0.832	0.215
Siebel et al. [113]	Total hip arthroplasty	PRO	71	36	35	CASPAR	C	0.914	0.547	0.132
Nishihara et al. [27]	Total hip arthroplasty	RCT	156	78	78	ROBODOC	BL, BT	0.999	0.873	0.237
Nakamura et al. [107]	Total hip arthroplasty	RCT	146	75	71	ROBODOC	C	0.998	0.851	0.224
Cobb et al. [106]	Unicompartmental knee arthroplasty	RCT	28	13	15	Acrobot	C	0.529	0.246	0.080
Park et al. [108]	Total knee arthroplasty	RCT	62	32	30	ROBODOC	C	0.872	0.490	0.121
Song et al. [29]	Total knee arthroplasty	RCT	60	30	30	ROBODOC	BL, C	0.861	0.478	0.119
Song et al. [30]	Total knee arthroplasty	RCT	100	50	50	ROBODOC	BL, C	0.977	0.697	0.168
Ringel et al. [77]	Spinal pedicle screw insertion	RCT	60	30	30	SpineAssist	OT, LOS	0.861	0.478	0.119
Total			10,147	5910	4237					

*BL* blood loss, *BT* blood transfusion rate, *OT* skin-to-skin operative (or procedure) time, *LOS* length of hospital stay, *C* 30-day overall complication rate, *CABG* coronary artery bypass grafting, *RCT* randomised controlled trial, *PRO* non-randomised prospective comparative studies

^a^Relevant to this review

^b^More than one comparison or study in an article

^c^Not computable, as there were more complications than the number of patients in the open group—complication data were excluded from meta-analysis as a result; for robotic studies on hips and knees, *n* = number of limbs

**Table 2:** Studies comparing robotic versus minimally invasive surgery

References	Procedure	Design	No. of patients, *n*			Robot	Perioperative outcomes addressed^a^	Power		
			Total	R	C			Effect size		
								Large	Medium	Small
Pigazzi et al. [56]	Rectal resection	PRO	12	6	6	dVSS	BL, LOS, C	0.241	0.123	0.061
Patriti et al. [55]	Rectal resection	PRO	66	29	37	dVSS	BL, BT, LOS, C	0.888	0.510	0.125
Baik et al. [89]	Rectal resection	PRO	113	56	57	dVSS	OT, LOS, C	0.984	0.726	0.176
Kim et al. [75]	Rectal resection	PRO	209	62	147	dVSS	BT, OT, LOS, C	1.000	0.908	0.260
Bertani et al. [16]^b^	Colectomy	PRO	64	34	30	dVSS	BL, LOS, C	0.882	0.502	0.123
Park et al. [51]	Colectomy	RCT	70	35	35	dVSS	BL, BT, LOS, C	0.910	0.541	0.131
Jiménez Rodríguez et al. [74]	Colorectal resection	RCT	56	28	28	dVSS	BT, LOS, C	0.836	0.451	0.114
Heemskerk et al. [102]	Rectopexy	PRO	33	14	19	dVSS	LOS	0.595	0.280	0.085
Wong et al. [57]	Rectopexy	PRO	63	23	40	dVSS	BL, LOS, C	0.853	0.468	0.117
Cadière et al. [97]	Fundoplication	RCT	21	10	11	Mona	LOS, C	0.412	0.193	0.072
Melvin et al. [84]	Fundoplication	PRO	40	20	20	dVSS	OT, C	0.693	0.338	0.095
Draaisma et al. [50]	Fundoplication	RCT	50	25	25	dVSS	BL, OT, LOS, C	0.791	0.410	0.107
Morino et al. [78]	Fundoplication	RCT	50	25	25	dVSS	OT, LOS, C	0.791	0.410	0.107
Nakadi et al. [99]	Fundoplication	RCT	20	9	11	dVSS	LOS, C	0.392	0.184	0.071
Lehnert et al. [15]	Fundoplication	PRO	20	10	10	dVSS	OT, C	0.395	0.185	0.071
Müller-Stitch et al. [98]	Fundoplication	RCT	40	20	20	dVSS	LOS, C	0.693	0.338	0.095
Hartmann et al. [101]	Fundoplication	PRO	80	18	62	dVSS	LOS, C	0.839	0.454	0.114
Sanchez et al. [100]	RYGB	RCT	50	25	25	dVSS	LOS, C	0.791	0.410	0.107
Benizri et al. [85]	RYGB	PRO	200	100	100	dVSS	OT, LOS, C	1.000	0.940	0.291
Mühlmann et al. [104]	Various bariatric^c^	PRO	20	10	10	dVSS	LOS, C	0.395	0.185	0.071
Park et al. [91]	Gastrectomy	PRO	150	30	120	dVSS	OT, LOS, C	0.973	0.682	0.164
Ruurda et al. [79]	Cholecystectomy	RCT	20	10	10	dVSS	OT	0.395	0.185	0.071
Nio et al. [105]	Cholecystectomy	PRO	20	10	10	ZRSS	LOS, C	0.395	0.185	0.071
Zhou et al. [52]	Cholecystectomy	RCT	40	20	20	ZRSS	BL, LOS, C	0.693	0.338	0.095
Kornprat et al. [90]	Cholecystectomy	PRO	46	20	26	ZRSS	OT	0.749	0.376	0.101
Berber et al. [58]	Liver resection	PRO	32	9	23	dVSS	BL, OT, C	0.504	0.234	0.078
Brunaud et al. [93]	Adrenalectomy	PRO	28	14	14	dVSS	OT, LOS, C	0.531	0.247	0.080
Morino et al. [81]	Adrenalectomy	RCT	20	10	10	dVSS	OT, LOS, C	0.395	0.185	0.071
Wu et al. [66]	Adrenalectomy	PRO	12	5	7	ZRSS	BL, LOS, C	0.236	0.121	0.061
Ploussard et al. [59]	Prostatectomy	PRO	288	83	205	dVSS	BL, BT, LOS, C	1.000	0.969	0.335
Gosseine et al. [67]	Prostatectomy	PRO	247	122	125	dVSS	BL, BT, LOS, C	1.000	0.975	0.347
Asimakopoulos et al. [72]	Prostatectomy	RCT	112	52	60	dVSS	BT, C	0.987	0.744	0.182
Porpiglia et al. [53]	Prostatectomy	RCT	120	60	60	dVSS	BL, OT, LOS, C	0.991	0.775	0.192
Khan et al. [17]^b^	Cystectomy	PRO	106	48	58	dVSS	BL, BT, OT, LOS, C	0.982	0.719	0.174
Caruso et al. [63]	Nephrectomy	PRO	20	10	10	dVSS	BL, BT, LOS, C	0.395	0.185	0.071
Hemal et al. [60]	Nephrectomy	PRO	30	15	15	dVSS	BL, BT, LOS, C	0.562	0.262	0.083
Kural et al. [62]	Nephrectomy	PRO	31	11	20	dVSS	BL, BT, LOS, C	0.540	0.251	0.081
Masson-Lecomte et al. [61]	Nephrectomy	PRO	265	220	45	dVSS	BL, BT, LOS, C	0.998	0.861	0.230
Bucerius et al. [18]^b^	CABG	PRO	97	24	73	dVSS	LOS	0.920	0.557	0.134
Mierdl et al. [92]	CABG	PRO	46	30	16	dVSS	OT, C	0.715	0.352	0.097
Sarlos et al. [54]	Hysterectomy—benign disease	RCT	95	47	48	dVSS	BL, OT, LOS, C	0.971	0.674	0.162
Paraiso et al. [73]	Hysterectomy—benign disease	RCT	52	26	26	dVSS	BT, OT	0.807	0.424	0.109
Hoekstra et al. [20]^b^	Endometrial cancer staging	PRO	39	32	7	dVSS	BL, OT, LOS, C	0.463	0.215	0.075
Jung et al. [19]^b^	Endometrial cancer staging	PRO	53	28	25	dVSS	BT, OT, LOS, C	0.814	0.430	0.110
Paraiso et al. [80]	Sacrocolpopexy	RCT	68	35	33	dVSS	OT, LOS, C	0.901	0.528	0.128
Seror et al. [64]	Sacrocolpopexy	PRO	67	20	47	dVSS	BL, LOS, C	0.839	0.454	0.114
Anger et al. [14]	Sacrocolpopexy	RCT	78	40	38	dVSS	BL, C	0.937	0.587	0.141
El Hachem et al. [65]	Various gynaecological—unspecified	PRO	91	39	52	dVSS	BL, LOS, C	0.962	0.646	0.154
Kolvenbach et al. [103]	AAA repair	PRO	39	8	31	ZRSS	LOS	0.502	0.233	0.078
Malcolme-Lawes et al. [21]^b^	AF ablation—robot 30 s	RCT	20	10	10	Sensei	BT, OT, C	0.395	0.185	0.071
Malcolme-Lawes et al. [21]^b^	AF ablation—robot 60 s	RCT^d^	20	10	10	Sensei	BT, OT, C	0.395	0.185	0.071
Steven et al. [83]	AF ablation	RCT	50	25	25	Sensei	OT, C	0.791	0.410	0.107
Kautzner et al. [87]	AF ablation	PRO	38	22	16	Sensei	OT, C	0.659	0.316	0.091
Di Biase et al. [86]	AF ablation	PRO	390	193	197	Sensei	OT, C	1.000	0.998	0.504
Steven et al. [82]	AF ablation	RCT	60	30	30	Sensei	OT	0.861	0.478	0.119
Tilz et al. [22]^b^	AF ablation—robot 30W	PRO	29	4	25	Sensei	OT	0.299	0.146	0.065
Tilz et al. [22]^b^	AF ablation—robot 20W	PRO	35	10	25	Sensei	OT	0.546	0.254	0.081
Rillig et al. [88]	AF ablation	PRO	70	50	20	Sensei	OT	0.846	0.461	0.116
Total			4301	1991	2310					

*BL* blood loss, *BT* blood transfusion rate, *OT* skin-to-skin operative (or procedure) time, *LOS* length of hospital stay, *C* 30-d overall complication rate, *RYGB* Roux-en-Y gastric bypass, *CABG* coronary artery bypass grafting, *AAA* abdominal aortic aneurysm, *AF* atrial fibrillation/flutter, *RCT* randomised controlled trial, *PRO* non-randomised prospective comparative study

^a^Relevant to this review

^b^More than one comparison or study in an article

^c^Gastric banding, implantable gastric stimulator, band revision

^d^Quasi-RCT (10 patients who underwent robotic AF ablation of 60-s duration were not randomized compared with 10 control patients that were randomised); for Baik 2009, *n* = 57 (control) for C and *n* = 51 (control) for OT and LOS, as 6 converted cases were excluded from analysis by authors; for Sarlos 2012, *n* = 47 (robotic) and *n* = 48 (control) for analysis of C, as no operations were performed in 5 patients, and for BL, OT, and LOS, *n* = 50 in each arm, as missing values were replaced with median of available measurements in respective study arm; for Mierdl 2005, *n* = 30 (robotic) for analysis of C but *n* = 24 for OT, as data not shown for 6 patients

#### Robotic versus OS

For robotic versus OS, there were 50 studies (11 RCTs and 39 prospective studies) (Table 1). The year of publication ranged from 1998 to 2013. In total, there were 5910 and 4237 patients in the robotic and OS groups, respectively. The smallest and largest sample sizes were 14 and 1738, respectively. The surgical robots used in these studies were the dVSS, Zeus^®^ Robotic Surgical System (ZRSS; Computer Motion Inc., Santa Barbara, California, USA), ROBODOC^®^ Surgical System (Curexo Technology Corp., Fremont, California, USA), Acrobot^®^ Surgical System (The Acrobot Co. Ltd., London, UK), CASPAR system (OrtoMaquet, Rastatt, Germany), and SpineAssist^®^ (Mazor Robotics Ltd., Caesarea, Israel).

#### Robotic versus MIS

For robotic versus MIS, there were 58 studies (21 RCTs and 37 prospective studies), which were published between 2001 and 2014 (Table 2). Taking into account all studies, the robotic and MIS groups consisted of 1991 and 2310 patients, respectively. Sample sizes ranged from 12 to 390. The surgical robots used were the dVSS, ZRSS, Mona (Intuitive Surgical), and Sensei^®^ Robotic Catheter System (Hansen Medical Inc., Mountain View, California, USA).

### Risk of bias assessment

**Fig. 3:**
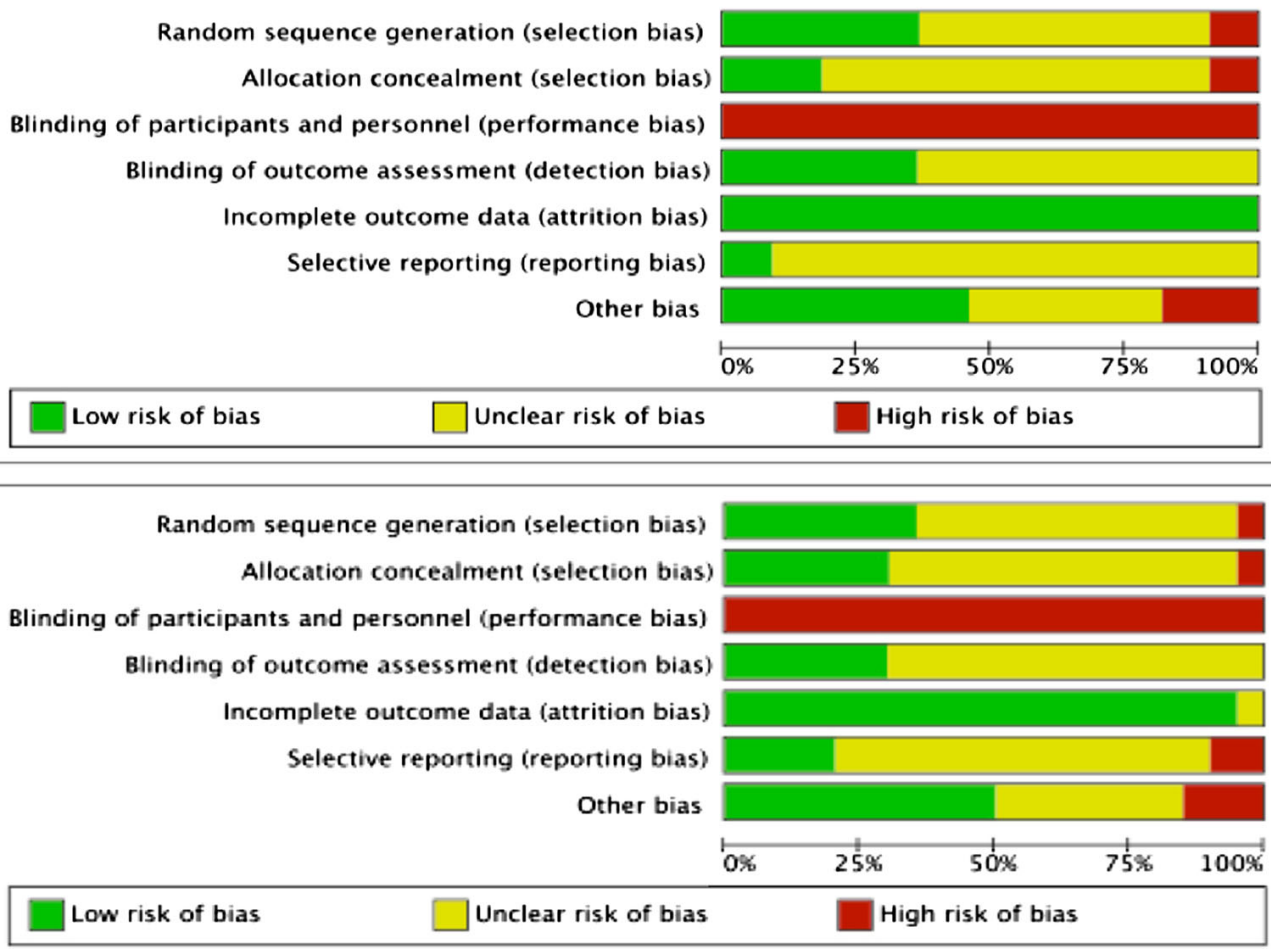
Risk of bias graphs of randomised controlled trials comparing robotic versus open surgery (above) and robotic versus minimally invasive surgery (below)

All included articles were assessed for the quality of their methodology. Of note, all 31 RCT articles suffered from a high risk of bias because they all showed a high risk of bias in the performance bias domain (Fig. 3). This was primarily due to the lack of surgeon blinding, which is unlikely to be possible in clinical trials of robotic surgery. As perioperative outcomes are especially vulnerable to performance bias, this risk was judged to be high. The subject of patient blinding, which is difficult in surgical trials but potentially feasible [24], was frequently unaddressed or unreported by authors. Most RCTs showed low risk of attrition bias, with complete perioperative outcome data. In many trials, however, the risk of bias related to sequence generation, allocation concealment, blinding of outcome assessor, and selective reporting was unclear, as sufficient information was not available due to poor reporting.

Of 68 articles of non-randomised prospective design, 55 (80.9 %) were of “higher” quality (Tables 3, 4). All prospective studies met the criteria for ascertainment of exposure, absence of outcome at the start of study, outcome assessment, and duration of follow-up. Most prospective studies also selected their control cohort from the same community as the robotic cohort and showed adequate follow-up. Many of them suffered from poor comparability, as expected from the lack of randomisation where selection bias is a caveat. In some cases, the representativeness of the robotic cohort in the community was felt not be adequate.

**Table 3:** Risk of bias of non-randomised prospective comparative cohort studies comparing robotic versus open surgery based on the Newcastle–Ottawa scale

References	Selection				Comparability	Outcome			Total score	
	Representativeness of robotic cohort	Selection of control cohort	Ascertainment of exposure	Absence of outcome at start of study		Assessment of outcome	Duration of follow-up	Adequacy of follow-up		
Menon et al. [34]	0	1	1	1	1	1	1	1	7	Higher
Bucerius et al. [18]^a^	0	1	1	1	1	1	1	1	7	Higher
Bach et al. [109]	0	1	1	1	1	1	1	1	7	Higher
Tewari et al. [36]	1	1	1	1	1	1	1	1	8	Higher
Siebel et al. [113]	0	1	1	1	1	1	1	1	7	Higher
Farnham et al. [31]	1	1	1	1	1	1	1	1	8	Higher
Rhee et al. [42]	0	1	1	1	0	1	1	1	6	Lower
Kiaii et al. [94]	1	1	1	1	1	1	1	1	8	Higher
Wood et al. [35]	1	1	1	1	1	1	1	1	8	Higher
Nelson et al. [95]	1	1	1	1	0	1	1	1	7	Higher
Ham et al. [37]	1	1	1	1	1	1	1	1	8	Higher
Poston et al. [43]	1	0	1	1	2	1	1	1	8	Higher
Ficarra et al. [32]	1	1	1	1	1	1	1	1	8	Higher
Hoekstra et al. [20]^a^	1	1	1	1	0	1	1	1	7	Higher
Lowe et al. [46]	0	1	1	1	1	1	1	1	7	Higher
Göçmen et al. [45]	0	1	1	1	1	1	1	1	7	Higher
Jung et al. [19]^a^	0	1	1	1	2	1	1	1	8	Higher
Carlsson et al. [111]	1	1	1	1	0	1	1	1	7	Higher
Hong et al. [38]	0	1	1	1	0	1	1	1	6	Lower
Doumerc et al. [69]	0	1	1	1	0	1	1	1	6	Lower
Kordan et al. [33]	1	1	1	1	0	1	1	1	7	Higher
Lee et al. [47]	1	1	1	1	2	1	1	1	9	Higher
Ng et al. [41]	1	1	1	1	1	1	1	1	8	Higher
Bertani et al. [16]^a^	1	1	1	1	2	1	1	1	9	Higher
Di Pierro et al. [70]	1	1	1	1	1	1	1	1	8	Higher
Kim et al. [112]	1	1	1	1	0	1	1	1	7	Higher
Martin et al. [40]	0	0	1	1	0	1	1	1	5	Lower
Balduyck et al. [110]	0	1	1	1	1	1	1	1	7	Higher
Kim et al. [68]	1	1	1	1	1	1	1	1	8	Higher
Khan et al. [17]^a^	0	1	1	1	0	1	1	1	6	Lower
Parekattil et al. [23]^a^	0	1	1	1	1	1	1	1	7	Higher
Bachinsky et al. [71]	0	1	1	1	1	1	1	1	7	Higher
Collins et al. [44]	0	1	1	1	2	1	1	1	8	Higher
Kim et al. [48]	1	1	1	1	2	1	1	1	9	Higher
Ryu et al. [96]	1	1	1	1	0	1	1	1	7	Higher
Ludovico et al. [39]	0	1	1	1	1	1	1	1	7	Higher
Masson-Lecomte et al. [49]	1	1	1	1	1	1	1	1	8	Higher

^a^Quality of articles with more than one study was assessed on their overall methodology

**Table 4:** Risk of bias of non-randomised prospective comparative cohort studies comparing robotic versus minimally invasive surgery based on the Newcastle–Ottawa scale

References	Selection				Comparability	Outcome			Total score	
	Representativeness of robotic cohort	Selection of control cohort	Ascertainment of exposure	Absence of outcome at start of study		Assessment of outcome	Duration of follow-up	Adequacy of follow-up		
Melvin et al. [84]	1	0	1	1	0	1	1	1	6	Lower
Bucerius et al. [18]^a^	0	1	1	1	1	1	1	1	7	Higher
Mühlmann et al. [104]	0	1	1	1	0	1	1	1	6	Lower
Brunaud et al. [93]	1	1	1	1	1	1	1	1	8	Higher
Nio et al. [105]	1	1	1	1	2	1	1	1	9	Higher
Kolvenbach et al. [103]	0	1	1	1	0	1	1	0	5	Lower
Mierdl et al. [92]	0	1	1	1	0	1	1	0	5	Lower
Pigazzi et al. [56]	0	1	1	1	0	1	1	1	6	Lower
Lehnert et al. [15]	1	1	1	1	0	1	1	1	7	Higher
Kornprat et al. [90]	0	1	1	1	2	1	1	1	8	Higher
Caruso et al. [63]	1	0	1	1	2	1	1	1	8	Higher
Heemskerk et al. [104]	0	1	1	1	1	1	1	1	7	Higher
Wu et al. [68]	1	1	1	1	1	1	1	1	8	Higher
Patriti et al. [57]	0	1	1	1	0	1	1	0	5	Lower
Baik et al. [91]	1	1	1	1	2	1	1	0	8	Higher
Hartmann et al. [102]	1	1	1	1	2	1	1	1	9	Higher
Ploussard et al. [59]	1	1	1	1	1	1	1	1	8	Higher
Gosseine et al. [67]	1	1	1	1	1	1	1	1	9	Higher
Hemal et al. [60]	1	1	1	1	2	1	1	1	9	Higher
Kural et al. [62]	0	1	1	1	2	1	1	1	8	Higher
Hoekstra et al. [20]^a^	1	1	1	1	0	1	1	1	7	Higher
Kautzner et al. [87]	0	0	1	1	0	1	1	1	5	Lower
Di Biase et al. [86]	1	1	1	1	2	1	1	1	9	Higher
Berber et al. [58]	1	0	1	1	2	1	1	1	8	Higher
Jung et al. [19]^a^	0	1	1	1	2	1	1	1	8	Higher
Tilz et al. [22]^a^	0	1	1	1	0	1	1	1	6	Lower
Bertani et al. [16]^a^	1	1	1	1	2	1	1	1	9	Higher
Wong et al. [57]	1	1	1	1	1	1	1	1	8	Higher
Kim et al. [75]	0	1	1	1	1	1	1	1	7	Higher
Park et al. [91]	0	1	1	1	2	1	1	1	8	Higher
Khan et al. [17]^a^	0	1	1	1	0	1	1	1	6	Lower
Seror et al. [64]	1	1	1	1	1	1	1	1	8	Higher
Rillig et al. [88]	0	1	1	1	2	1	1	1	8	Higher
Benizri et al. [85]	0	1	1	1	1	1	1	1	7	Higher
Masson-Lecomte et al. [61]	1	1	1	1	0	1	1	1	7	Higher
El Hachem et al. [65]	1	1	1	1	1	1	1	1	8	Higher

^a^Quality of articles with more than one study was assessed on their overall methodology

### Meta-analyses of perioperative surgical outcomes

(i) Blood loss

#### Robotic versus OS

**Fig. 4:**
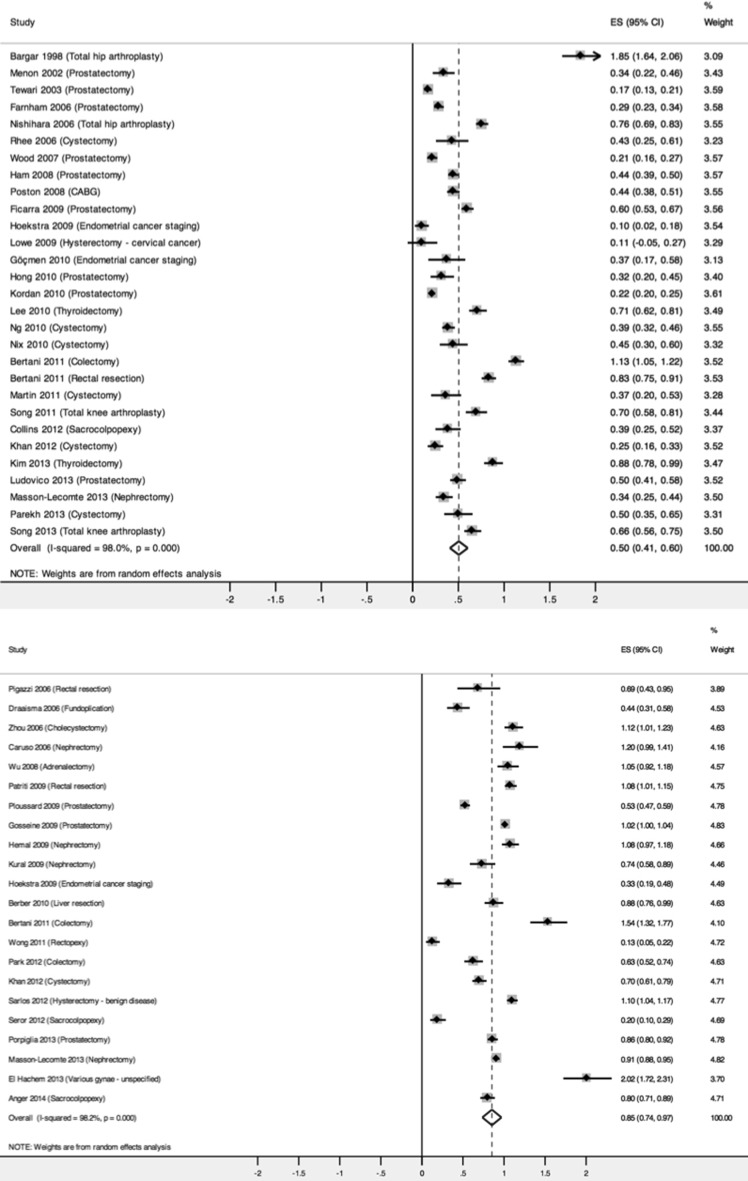
Forest plots of blood loss; robotic versus open surgery (above), robotic versus minimally invasive surgery (below)

There were six RCT [25–30] and 23 prospective [16, 17, 20, 31–49] studies reporting on blood loss, giving a total of 29 studies overall. Meta-analysis demonstrated blood loss in the robotic arm to be 50.5 % of that in the OS arm (Fig. 4). This reduction was significant (95 % CI 0.408–0.602). There was high heterogeneity in the results (*I*^2^ = 98.0 %). Sensitivity analysis on RCTs showed reduction in blood loss, but this was no longer significant (pooled RoM: 0.807, 95 % CI 0.563–1.051, *I*^2^ = 96.3 %).

#### Robotic versus MIS

Twenty-two studies reported blood loss as an outcome measure. Of these, six were RCT studies [14, 50–54] and 16 were prospective studies [16, 17, 20, 55–67]. Meta-analysis of these studies confirmed a significant reduction in blood loss in favour of robotic surgery, which was 85.3 % of that experienced by patients in the MIS arm (95 % CI 0.736–0.969) (Fig. 4). The heterogeneity was high (*I*^2^ = 98.2 %). Sensitivity analysis performed on RCTs and, however, revealed a non-robust result (pooled RoM: 0.830, 95 % CI 0.653–1.008, *I*^2^ = 95.9 %).

(ii) Blood transfusion rate

#### Robotic versus OS

Blood transfusion rate was investigated in two RCT [26, 27] and 16 prospective [17, 19, 31–36, 38, 42, 45, 49, 68–71] studies. Forty-two of 2127 patients (2.0 %) in the robotic group needed blood transfusion compared with 249 of 1869 patients (13.3 %) in the open group. One study [27] was excluded from quantitative synthesis, as its effect size was not computable. Meta-analysis of the remaining 17 studies demonstrated the risk of blood transfusion with robotic surgery to be 27.2 % of that of OS. This reduction in favour of robotic surgery was significant (95 % CI 0.165–0.449). The results showed moderate heterogeneity (*I*^2^ = 55.2 %). Sensitivity analysis on RCTs was not done, as only one study was available. In this RCT, no significant difference in blood transfusion requirement was demonstrated (RR 0.800, 95 % CI 0.400–1.600) [26].

#### Robotic versus MIS

Six RCT [21, 51, 72–74] and ten prospective [17, 55, 59–63, 67, 75] studies reported blood transfusion requirement. Taking all these studies together, 4.2 % (33/789) of patients who underwent robotic intervention compared with 6.5 % (56/856) of MIS patients received blood transfusion. Computation of valid RR was not possible in three studies [21, 51, 63], hence their exclusion from meta-analysis. From the remaining 13 studies, we demonstrated a significant reduction in the requirement for blood transfusion in patients who underwent robotic surgery compared with MIS (pooled RR 0.621, 95 % CI 0.390–0.988). The heterogeneity was low (*I*^2^ = 0.0 %). Nevertheless, the result of sensitivity analysis on RCTs was inconsistent (pooled RR 1.329, 95 % CI 0.325–5.438, *I*^2^ = 0.0 %).

(iii) Operative time (skin-to-skin)

#### Robotic versus OS

Sixteen studies assessed operative time. These comprised three RCT [26, 76, 77] and 13 prospective [17, 19, 20, 23, 34, 36, 41, 42, 45–47, 49] studies. Meta-analysis showed robotic surgery to increase operative time by 7.3 %, which was significant (95 % CI 1.022–1.124). High heterogeneity was found (*I*^2^ = 91.8 %). Sensitivity analysis on RCTs showed a consistent result (pooled RoM: 1.162, 95 % CI 1.016–1.308, *I*^2^ = 86.8 %).

#### Robotic versus MIS

Operative time was investigated by 12 RCT [21, 50, 53, 54, 73, 78–83] and 18 prospective [15, 17, 19, 20, 22, 58, 75, 84–93] studies. There was a significant prolongation of operative time by 13.5 % over MIS when surgical robots were utilised (95 % CI 1.096–1.173). Heterogeneity was high (*I*^2^ = 92.3 %). When only RCTs were considered in a sensitivity analysis, the result remained robust (pooled RoM: 1.202, 95 % CI 1.119–1.286, *I*^2^ = 87.1 %).

(iv) Length of hospital stay

#### Robotic versus OS

Thirty studies compared length of hospital stay between robotic and open interventions. There were 4 RCT [25, 26, 28, 77] and 26 prospective [16–20, 32, 34–37, 39–43, 45–47, 49, 68, 69, 71, 94–96] studies. The result for one study [26] was not computable. Meta-analysis of the remaining 29 studies revealed length of stay for patients who underwent robotic surgery to be 69.5 % of those who underwent OS. This decrease was significant (95 % CI 0.615–0.774). Heterogeneity was high (*I*^2^ = 98.5 %). In contrast, when only RCTs were considered, the improvement in length of stay was lost (pooled RoM: 1.038, 95 % CI 0.878–1.197, *I*^2^ = 89.4 %).

#### Robotic versus MIS

Length of hospital stay was addressed by 40 studies, of which 13 were RCT [50–54, 74, 78, 80, 81, 97–100] and were prospective [16–20, 55–57, 59–66, 75, 85, 89, 91, 101–105] studies. Ten studies [16, 20, 50, 52, 57, 91, 97, 100, 104, 105] were excluded from meta-analysis, as their effect sizes were not computable. Meta-analysis of the remaining 30 studies showed no significant difference in duration of stay (pooled RoM: 0.982, 95 % CI 0.936–1.027). High heterogeneity was noted (*I*^2^ = 93.4 %). Sensitivity analysis on RCTs remained robust (pooled RoM: 1.001, 95 % CI 0.955–1.047, *I*^2^ = 80.2 %).

(v) Overall complication rate (30 day)

#### Robotic versus OS

Overall complications were compared in nine RCT [25, 26, 28–30, 76, 106–108] and 28 prospective [16, 17, 19, 20, 32, 34–37, 39, 41, 43, 45–47, 49, 68–70, 94–96, 109–113] studies. From these studies, the overall complication rate was 11.6 % (515/4453) in the robotic arm compared with 21.4 % (693/3245) in the open arm. Results from three studies [29, 96, 109] did not allow for computable RRs. From the remaining 34 studies, meta-analysis demonstrated a significant decrease in overall complication rate in favour of robotic surgery, which was 63.7 % of that with OS (95 % CI 0.483–0.838). High heterogeneity was present (*I*^2^ = 81.9 %). Sensitivity analysis on RCTs was, however, inconsistent with the primary analysis (pooled RR 1.090, 95 % CI 0.631–1.881, *I*^2^ = 59.9 %).

#### Robotic versus MIS

Forty-eight studies investigated complications. There were 18 RCT [14, 21, 50–54, 72, 74, 78, 80, 81, 83, 97–100] and 30 prospective [15–17, 19, 20, 55–66, 75, 84–87, 89, 91, 92, 101, 104, 105] studies. Taking all these studies into consideration, the overall complication rate in the robotic arm was 16.1 % (288/1789) compared with 15.7 % (317/2025) in the MIS arm. Valid effect sizes in the form of RR were not producible from results of nine studies [15, 52, 66, 78, 83, 84, 87, 104, 105]. Meta-analysis involving the remaining 39 studies demonstrated no significant difference in overall complication rate between robotic and MIS (pooled RR 0.988, 95 % CI 0.822–1.188). Heterogeneity was low (*I*^2^ = 23.0 %). When sensitivity analysis was performed on RCTs, the result remained robust (pooled RR 1.187, 95 % CI 0.851–1.654, *I*^2^ = 15.4 %).

**Fig. 5:**
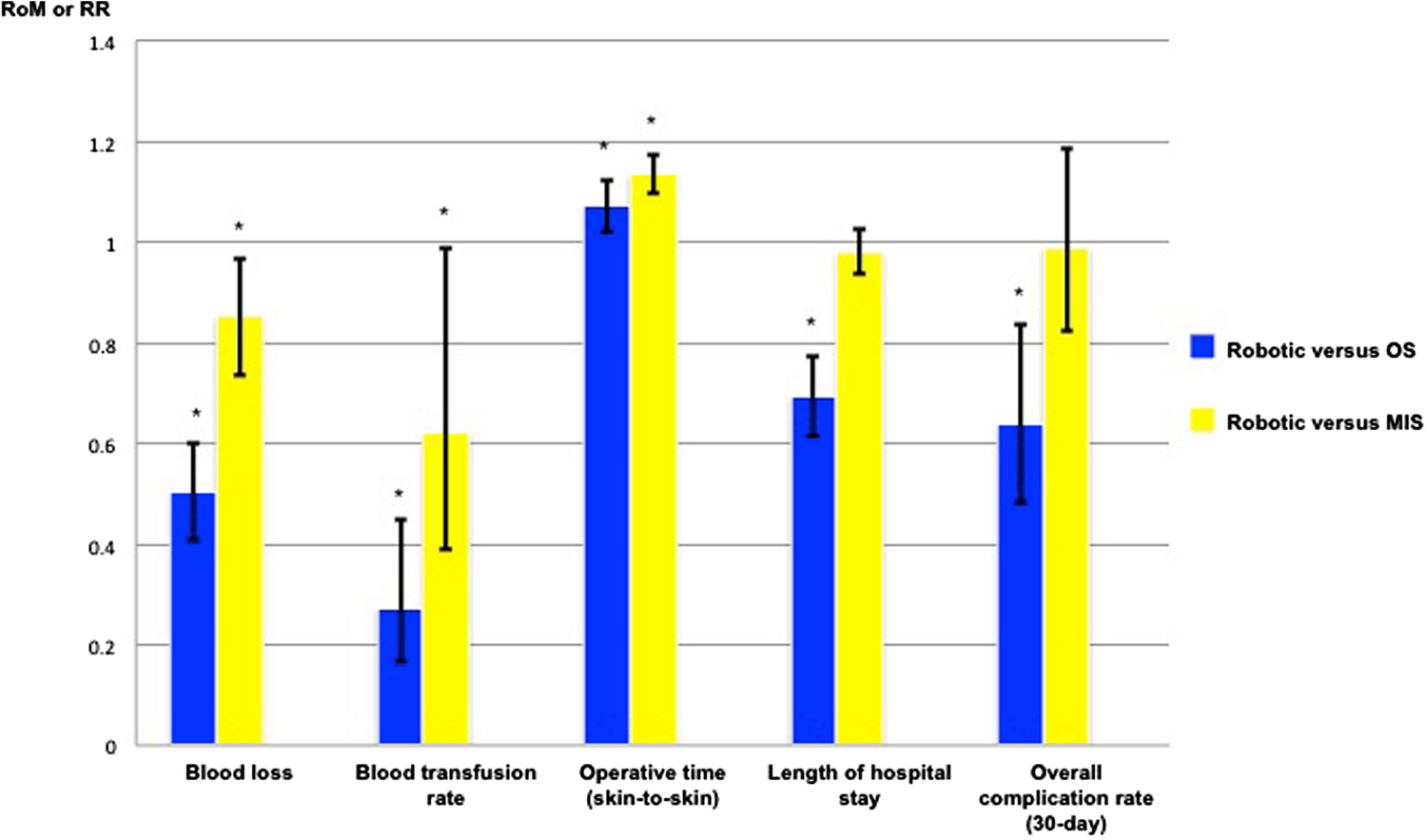
Pooled proportional change in perioperative outcomes for robotic versus open surgery and robotic versus minimally invasive surgery, with 95 % confidence interval. *RoM* ratio of means, *RR* risk ratio, *OS* open surgery, *MIS* minimally invasive surgery. *Significant effect

Results of our meta-analyses are summarised in Fig. 5.

### *Post hoc* power analyses

With respect to RCT studies, for large effect sizes, just 17 [14, 27–30, 51, 53, 54, 72–74, 76, 77, 80, 82, 107, 108] of 32 studies (53.1 %) had adequate statistical power (that is, power >80 %). This fell to four studies [27, 28, 76, 107] (12.5 %) for medium effect sizes. For small effect sizes, no RCT study had adequate power.

Analysis of the 76 prospective studies revealed that just 47 [16–20, 23, 31–37, 39, 41, 43, 47, 49, 55, 57, 59, 61, 64, 65, 67–71, 75, 85, 86, 88, 89, 91, 94–96, 101, 111–113] of them (61.8 %) had adequate power for outcome evaluation, assuming large effect sizes. For medium effect sizes, 20 studies [31–33, 35–37, 41, 43, 59, 61, 67–70, 75, 85, 86, 95, 111, 112] (26·3 %) were sufficiently powered. Only three studies [33, 95, 111] (4.2 %) had adequate power for small effect sizes.

The lack of statistical power in many studies is not surprising given that in only 16 RCT (50 %) and six prospective (7.9 %) studies were primary outcomes clearly defined and a priori power analysis performed (Table 5). Furthermore, only a handful of these studies [51, 54, 73, 80, 82, 85] were powered to the outcomes investigated in this review.

**Table 5:** Studies with clearly defined primary outcomes and where power analysis was undertaken a priori

Study	Procedure	Design	Primary outcome						
			OT	LOS	C	Onc	Func	Cost	Other
*Robot versus MIS*									
Draaisma et al. [50]	Fundoplication	RCT							✔ Barium swallow, manometry, ph study
Morino et al. [78]	Fundoplication	RCT						✔	
Steven et al. [83]	AF ablation	RCT							✔ Radiofrequency duration
Steven et al. [82]	AF ablation	RCT	✔						
Asimakopoulos et al. [72]	Prostatectomy	RCT					✔		
Paraiso et al. [80]	Sacrocolpopexy	RCT	✔						
Park et al. [51]	Colectomy	RCT		✔					
Sarlos et al. [54]	Hysterectomy—benign disease	RCT	✔						
Porpiglia et al. [53]	Prostatectomy	RCT					✔		
Paraiso et al. [73]	Hysterectomy—benign disease	RCT	✔						
Benizri et al. [85]	Roux-en-Y gastric bypass	PRO			✔				
El Hachem et al. [65]	Various gynae—unspecified	PRO							✔ Pain
Anger et al. [14]	Sacrocolpopexy	RCT						✔	
*Robot versus OS*									
Cobb et al. [106]	Unicompartmental knee arthroplasty	RCT							✔ Leg alignment
Wood et al. [35]	Prostatectomy	PRO							✔ Quality of life
Nix et al. [25]	Cystectomy	RCT				✔			
Hong et al. [38]	Prostatectomy	PRO							✔ Venous gas embolism
Song et al. [29]	Total knee arthroplasty	RCT							✔ Leg alignment
Ringel et al. [77]	Spinal pedicle screw insertion	RCT							✔ Implant position
Collins et al. [44]	Sacrocolpopexy	PRO							✔ Return to baseline activity (accelerometer)
Song et al. [30]	Total knee arthroplasty	RCT							✔ Leg alignment
Kim et al. [48]	Thyroidectomy	PRO							✔ Intraocular pressure

Note absence of outcome for blood loss and blood transfusion

*OT* operative time (includes fluoroscopy time), *LOS* length of stay, *C* complication, *Onc* oncological (includes lymph node yield), *Func* functional (includes erectile function, continence), *RYGB* Roux-en-Y gastric bypass, *AF* atrial flutter/fibrillation, *RCT* randomised controlled trial, *PRO* non-randomised prospective comparative study

Results of post hoc power analyses for individual studies are presented in Tables 1 and 2.

## Discussion

The term “disruptive innovation” represents a process where a product establishes itself at the bottom of a market and climbs through this sector to displace competitors [114]. Initial characteristics of a disruptive innovation model include: (i) simpler products and services, (ii) smaller target markets, and (iii) lower gross margins. As a result, these innovations can “create space” at the bottom of the market to allow new disruptive competitors to emerge. Currently in the field of robotic surgery, the promise of simplicity has yet to be translated into daily practice. Furthermore, the evidence regarding cost efficacy and gross margins has been poorly documented so that decisions regarding the adoption of robotic surgery remain controversial.

However, to disregard robotic surgery completely as an unfulfilled promised in its 30 years of existence may be imbalanced. Our meta-analyses of all RCTs and prospective studies to date, regardless of specialty and procedure type, revealed a decrease in blood loss and blood transfusion rate with robotic surgery when compared with both OS and MIS. Additionally, comparison against OS demonstrated a reduction in length of hospital stay and overall complication rate in favour of robotic surgery.

The ability of robotic surgery to reduce blood loss and need for blood transfusion may be attributed to its advanced features, which could improve surgical precision. This would be important in avoiding injury to vessels and other structures that can cause unintended bleeding. The additional benefits of robotic surgery over OS, in the form of shorter length of hospital stay and fewer complications, may partly be due to its capacity for minimal access. These benefits have been demonstrated in conventional minimally invasive surgical procedures [115–118], where the positive effect of reduced tissue trauma has been implicated [118]. Given its added features, the inability of robotic surgery to achieve improved length of stay and complication rate over MIS can be considered surprising. This may be reflective of the status that surgical robots have not yet exceeded their effects beyond those of conventional minimally invasive platforms for these outcomes. Alternatively, these outcomes may be inadequate markers for accurately capturing the increased precision of robotic surgery. More sensitive assessment tools of precision are advocated in future trials, which might include video appraisal of intraoperative tissue handling, errors, and efficiency [52, 105].

When RCTs were analysed separately, the proportional benefits of robotic surgery were lost. Given their higher level of evidence, these RCTs may be considered as more representative of the true population effect, although they are limited by a profound lack of numbers. We identified only 31 clinical RCTs on robotic surgery, which is a fraction (0.1 %) of the 28,574 potentially relevant articles. Many RCTs failed to clearly define primary outcomes and perform a priori power analysis, which led to inadequate sample sizes and hence, statistical power necessary for outcome evaluation. Through post hoc analyses, we showed that just over half of all RCTs were adequately powered to detect a true difference in outcomes for large effect sizes. For smaller effect sizes, this deficiency, inevitably, was further amplified. These findings are probably related to common barriers in undertaking successful surgical RCTs, including ethical issues, challenging patient recruitment and randomisation due partly to lack of equipoise, learning curve, inexperience in designing trials, inadequate medical statistical knowledge, problematic long-term follow-up, and insufficient funding and resources [24, 119]. Furthermore, difficulty in blinding is a major methodological barrier [120, 121]. Consequently, all included RCTs were considered to suffer from a high risk of performance bias, and accordingly, a high risk of bias overall [7]. Together, these factors could explain the non-robust results.

The demonstration of longer operative time with robotic surgery contradicts its proposed aims of facilitating operative tasks that would otherwise be difficult to perform efficiently with conventional tools. One possible explanation is the requirement for additional steps in their deployment. For example, docking is needed for surgical robots such as the dVSS [73, 80]. Hardware issues could also explain the longer operative time, as surgical robotic instruments may be cumbersome to place or switch efficiently, or may be insufficiently adapted for the specific purpose [78, 80, 81, 97].

The surgical learning curve has implications on our findings. Before study commencement, individual surgeons have typically performed far fewer robotic cases than conventional ones [51, 53, 54, 72, 73, 107]. This disparity could disadvantage robotic surgery due to relatively less familiarity. This could further explain the prolonged operative time of robotic surgery. Nevertheless, our demonstration of at least equivalent outcomes for other perioperative variables may be regarded as a favourable effect of robotic surgery. By allowing achievement of similar or better outcomes despite the relative lack of user experience, surgical robots may be important in facilitating training and attainment of competences. Furthermore, many surgeons may view surgical robots as an “enabling technology”, without which it would not be possible for them to perform certain complex minimally invasive procedures [122]. Pure laparoscopic radical prostatectomy, which demonstrates significant technical challenges, is an example of a procedure where robotic assistance in suturing and other laparoscopic tasks is important [123]. Although robotic surgery needs to demonstrate more than just equivalent patient outcomes to be cost-effective due to its substantial costs, its potential positive effects on surgeon ability must also be considered.

This systematic review has some limitations. Our focus on blood loss, blood transfusion rate, operative time, length of hospital stay, and complications was based primarily on the fact that these were the most commonly reported outcomes in the robotic surgery literature. However, these standard parameters may not fully demonstrate the true value of robotic surgery, especially when the overall benefits are not always clearly perceptible in the short term. Utilisation of dedicated research parameters should be encouraged [124]. Already, there is an increasing inclination towards such parameters that are probably more relevant, including functional, oncological, and quality of life outcomes, specific anatomical–pathological endpoints (such as nerve damage control), and ergonomics. With continuing improvement in outcome parameter selection by clinical research teams, future evidence synthesis centred on these parameters may better reflect the added value of robotic surgery.

Our appraisal of robotic surgery through an exclusively clinical viewpoint has also meant that other elements of innovation evaluation could not be incorporated into our conclusions. These include the impact of surgical robotics on intellectual property and patent generation, resource management, healthcare leadership, mentorship, training, cost efficacy, marketing strategy, business strategy, and stakeholder value generation.

When meta-analyses were possible, the heterogeneity was frequently high. However, this is not unexpected given the wide variability in patient cohorts and interventions. There was additional variability within specific procedures. For instance, Nissen [50, 78, 84, 97–99], Toupet [84], Dor [101], and Thal [15] fundoplications were variant techniques performed in different studies. Furthermore, the extent of robotic assistance varied from its utilisation in anastomotic suturing only [103] to totally robotic procedures [21, 22, 82, 83, 85–88, 92, 100]. Methodological diversity in the form of different study designs and risks of bias also contributed to the heterogeneity.

We incorporated different surgical robots in our review, including those that are no longer in use, such as the ZRSS. However, our intention was not to compare outcomes of specific procedures obtainable through currently available robots but to evaluate, via an overview of commonly addressed perioperative outcomes, whether the goals of robotic surgery in general have been achieved. Hence, we offered a unique perspective on robotic surgery by covering the 30 years of its existence. Accordingly, we also elected not to stratify our analysis based on robot or procedure type. Consequently, this restricts the applicability of this review, so that the individual stakeholder interested in outcomes for a specific intervention may not be able to draw sufficiently relevant evidence from our results.

Prospective studies were included to address the paucity of RCTs. Although practical, their inclusion inevitably introduces other biases associated with this study design. Moreover, caution is advised in the interpretation of complication data, as there were inconsistencies in their reporting. Many authors failed to comply with the quality criteria [125] for complication reporting. There was also a lack of agreement in terms of what constitutes complications, such as with regard to blood transfusion and conversion. Nevertheless, this issue is not unique to our included studies [126, 127]. Additionally, studies on robotic surgery continue to suffer from several methodological flaws, including a lack of studies that offer multiple endpoint analysis [128] in such a complex field.

The Society of American Gastrointestinal and Endoscopic Surgeons [122] and European Association of Endoscopic Surgeons [124] consensus statements on robotic surgery have also highlighted the lack of high-quality data in evaluating the health outcomes of this technology. Upcoming research efforts should improve on current methodological deficiencies. The implementation of outcome registries for robotic surgery is important to document and compare benefits and harms and in identifying the direction for future development [122]. More robust controlled trials should be undertaken, particularly in areas where robotic surgery has shown some potential, such as complex hepatobiliary surgery, bariatric and upper gastrointestinal revisional surgery, gastric and oesophageal cancer surgery, rectal surgery, and surgery for large adrenal masses [124].

## Conclusions

After the promising pioneering clinical application of PUMA 560 in 1985, the stage was set for robotic surgery to assume the role of a significant disruptive innovation in health care. Three decades on, our analysis across a wide range of surgical robots identified their overall positive contribution in reducing blood loss and blood transfusion rate over OS and MIS. Additionally, against OS, they showed overall proportional improvement in length of hospital stay and overall complication rate. These beneficial effects were lost when only RCTs were appraised, although these RCTs were themselves limited. Longer operative time was a common caveat. Further well-conducted surgical trials are needed to confirm these findings. Whilst the barriers for these trials may seem insurmountable, solutions to overcoming them are now increasingly recognised. These may involve ensuring protocol transparency, improving trial dissemination, creating specialised trial units, establishing dedicated outcome monitoring groups, implementing appropriate minimum surgeon experience to reduce the impact of learning curves, and incorporating research training in the surgical curriculum [119]. To ensure better outcomes for future robotic surgery, a multidisciplinary approach during product development involving close collaboration between surgeons and engineers, in addition to inclusive patient engagement, is mandatory. With the advent of more affordable, enriching technologies can be modularly incorporated into conventional surgical approaches such as intraoperative fluorescence imaging, high-definition 3-D visualisation, wristed endoscopic hand tools, and navigation systems, robotic surgery risks degenerating into an unfulfilled promise if it fails to innovate in line with stakeholders’ needs.
